# Autonomic regulation and comorbid symptoms in children with attention deficit hyperactivity disorder

**DOI:** 10.1007/s00702-024-02832-9

**Published:** 2024-09-11

**Authors:** Kira Kehm, Susan Schloß, Christopher Mann, Katja Becker, Udo König, Wilfried Pott, Mira-Lynn Chavanon, Ursula Pauli-Pott

**Affiliations:** 1 Department of Child and Adolescent Psychiatry, Psychosomatics and Psychotherapy, Philipps-University of Marburg, Schützenstrasse 45, D-35039 Marburg, Germany; 2 Department of Psychology, Philipps-University, Marburg, Germany; 3 Center for Mind, Brain and Behavior (CMBB), University of Marburg and Justus Liebig University, Giessen, Germany

**Keywords:** ADHD, Heart rate variability, Autonomic nerve system, Internalizing symptoms, Callous unemotional traits, Low prosocial emotionality

## Abstract

**Objective:**

Vagally-mediated heart rate variability (vmHRV) is regarded as transdiagnostic marker of emotion regulation and cognitive control capacity. We analysed vmHRV of children with attention deficit/hyperactivity disorder (ADHD). Based on previous research, we expected to find comorbid symptom dimensions (i.e. internalizing symptoms, conduct problems (CP), and callous unemotional (CU) traits) to relate to vmHRV measures.

**Methods:**

The sample comprised 100 (70 boys) medication naïve children with ADHD. Children were 6 to 11 years old. High frequency HRV (HF-HRV) was measured at rest and during a delay of gratification task. Additionally, sympathetic reactivity was assessed via skin conductance responses (SCR). Comorbid symptoms were assessed by parent-report questionnaires and clinical interviews.

**Results:**

The multiple correlation between CU traits and the HF-HRV scores proved statistically significant. Higher CU traits were associated with higher HRV resting-state and response scores. CP were positively associated with the SCR score.

**Conclusion:**

In children with ADHD, increased CU traits might point to a comparatively less impaired self-regulation capacity in the reward-related context. The result corresponds to findings from previous studies. In the future, CU traits should be considered in analyses of autonomic regulation in ADHD.

## Introduction

During the last decades heart rate variability (HRV) has become a marker of flexible adaptation to environmental demands, and proved to be associated with health and successful adjustment outcomes (Beauchaine and Thayer 2015; Thayer et al. 2012). The autonomic nervous system (ANS) controls heart rate (HR) and the variability between consecutive heart beats, the so-called HRV. Both branches of the ANS, namely the sympathetic and parasympathetic system, contribute to its regulation. As sympathetic effects take much longer to unfold than parasympathetic effects, parasympathetic influences primarily modulate cardiac beat to beat activity (Shaffer and Ginsberg 2017) and specific parameters of the HRV are assumed to reliably estimate primarily parasympathetic (vagal) regulation (vagally-mediated HRV, vmHRV, Thayer et al. 2012 ).

Consistent evidence has been found for associations of vmHRV with measures of emotion regulation, attentional capacity, and cognitive control (Holzman and Bridgett 2017; Wagner and Waller 2020). A meta-analysis by Thayer et al.(2012) revealed that vmHRV relates to the activity of the prefrontal cortex (PFC) during top-down control over behavior. Given this background, studies analysed associations between altered vmHRV and mental disorders characterized by poor cognitive control and emotion regulation capacity. Some evidence was found for a relationship between vmHRV (measured during resting state and in response to emotion-eliciting stimuli) and several internalizing and externalizing disorders (Beauchaine 2015). In this context, vmHRV was analysed in children with attention deficit/hyperactivity disorder (ADHD).

ADHD is an externalizing disorder characterized by symptoms of inattention, hyperactivity, and impulsivity. Children with ADHD, display deficits in core executive functions such as inhibitory control, increased irritability and emotion dysregulation (Shaw et al. 2014; Pauli-Pott and Becker 2015; Hartman et al. 2019; Nigg 2022). However, a recent meta-analysis found no association between resting state vmHRV and ADHD (Koenig et al. 2017). Overall, children with ADHD did not differ from healthy children in resting-state vmHRV. Effect sizes of the primary studies were heterogeneous. A further meta-analysis (Robe et al. 2019) focused on task-related HRV (i.e. changes of HRV in response to attention- and emotion-regulation tasks) and revealed a small, significant effect. Children with ADHD showed lower vmHRV responses than healthy children. Effect sizes of the primary studies were heterogeneous and moderator analyses revealed a larger effect for children with comorbid conduct disorders. A reason for the small and heterogeneous mean effect sizes might lie in the heterogeneity of the disorder. ADHD represents a highly heterogeneous disease, not just with respect to the etiological pathways but also regarding the presentation of core symptoms, comorbid symptoms, cognitive and emotional traits (Pacheco et al. 2022; Nigg et al. 2020).

Some children with ADHD show increased negative emotionality/ irritability and symptoms of internalizing disorders, and approximately 25% of children with ADHD develop comorbid depressive disorders (Faraone et al. 2015). Low vmHRV is associated with anxiety (Friedman 2007) and depressive disorders (Koch et al. 2019; Schiweck et al. 2019; Koenig et al. 2016). Hence, it is possible that vmHRV activity and reactivity in ADHD is dependent on the extent of comorbid anxious/depressive symptoms (i.e. internalizing symptoms).

Many children with ADHD show conduct problems (i.e. symptoms of oppositional defiant disorder, ODD, and/or conduct disorder, CD) and about 50% develop full-blown ODD and/or CD (Faraone et al. 2015). Moreover, children with ADHD may show callous-unemotional (CU) traits (Blair et al. 2014), a clinical specifier in CD. CU traits include low prosocial emotionality, low empathy, lack of remorse or guilt, shallow or deficient affect and a lack of concern about performance. CU traits are frequent in children with ODD, CD, and ADHD (Blair et al. 2014) and appear in children not showing these disorders (Fanti and Kimonis 2017).

As vmHRV might indicate adequate emotion regulation in the social context, it was hypothesized that children with CU traits would show reduced baseline vmHRV and vmHRV reactivity to emotional cues (Wagner and Waller 2020). Individual study results, however, are mixed. Musser et al. (2013) found children with ADHD and low prosocial behavior to show low sympathetic and parasympathetic activity and reactivity in comparison to healthy children. In contrast, a study by Thomson et al. (2020) yielded an association between high CU traits and heightened sympathetic and parasympathetic co-activation during fear induction in adolescents with externalizing disorders (50% with ADHD). The pattern might be indicative of an increased capacity to maintain control while experiencing fear (Thomson et al. 2020). Likewise, Goulter et al. (2019) found high vmHRV activity and reactivity to be associated with psychopathic traits in a sample of young woman. A meta-analysis by Fanti et al. (2019) found no association between CU-traits and heart rate (re-)activity in children and adolescents with conduct problems (i.e. ODD, CD, including children with comorbid ADHD), but, only two studies have examined this issue.

To summarize, vmHRV represents a transdiagnostic marker of arousal/emotion regulation and adaptation to environmental demands. ADHD is a highly heterogeneous disease with large individual differences in emotional and cognitive functions. In children with ADHD, vmHRV activity and reactivity might covary with internalizing symptoms, conduct problems, and/or CU traits. In the present study, we adopt a dimensional measurement approach to account for the whole spectrum of symptoms. We aim to analyse whether resting-state and task-related vmHRV relate to comorbidity dimensions in a sample of psychotropic-medication naïve children with ADHD. We expect to find associations of resting-state and task-related vmHRV with (1) conduct problems, (2) CU-traits and (3) internalizing symptoms.

For comparison and validation purposes, we descriptively assess associations between these comorbidity dimensions and the sympathetic reactivity and compare the ADHD group with a small sample of healthy children.

## Methods

### Participants

The study sample consisted of 100 (70 boys) children, aged between 6 and 11 years, and diagnosed with ADHD. Children were psychotropic medication-naïve (i.e. never received any psychopharmacological treatment). Further exclusion criteria were: IQ < 80, psychiatric diagnosis of autism, motor or sensory disability, chronic physical diseases involving brain functions, any continuous pharmacological treatment in the last three months, and insufficient German language skills of parents or child. Children were recruited via a child and adolescent psychiatry practice and outpatient clinics in Gießen, Marburg, and Butzbach (Middle Hesse, Germany). A sample of 24 healthy children was recruited through primary and secondary schools of the same district. In addition to the exclusion criteria used for the ADHD group, children with a diagnosis of a mental disorder were excluded.

The ADHD diagnostic module of the Child and Adolescent Psychiatric Interview (CAPA) by Angold et al. (1995) in the German-language DSM-5 version (translated by Dr. Yvonne Otto, Child and Adolescent Psychiatric Clinic, University of Leipzig) was conducted with the mothers of all children. The CAPA is a well-validated, widely established clinical interview that allows clinical diagnoses to be made according to the DSM-5. Table 1 contains the descriptive data of the samples. Parents and children gave their written informed consent to participate in the study and received an expense allowance of 30 Euros. The study was approved by the Ethics Committee of the Medical Faculty, University of Marburg.

**Table 1:** Description of samples

	Children with ADHD *n* = 100	Healthy children *n* = 24	Statistical tests^a^
Sex	n (%)	n (%)	Chi^2^ (1) = 0.50 ns
male	70 (70.0)	15 (62.5)	
female	30 (30.0)	9 (37.5)	
Age in months	m (s) 108.28 (20.09)	m (s) 113.39 (20.66)	t (122) = 1.09 ns
Education level of mother	n (%)	n (%)	Chi^2^ (3) = 3.79 ns
no compl./ basic education	21 (21.4)	1 (4.3)	
vocational qualification	31 (31.6)	8 (34.8)	
high school	24 (24.5)	7 (30.4)	
university	22 (22.4)	7 (30.4)	
(no reply)	2	1	
Education level of father	n (%)	n (%)	Chi^2^ (3) = 6.55 ns
no compl./ basic education	30 (31.3)	4 (18.2)	
vocational qualification	35 (36.5)	5 (22.7)	
high school	10 (10.4)	6 (27.3)	
university	21 (21.9)	7 (31.8)	
(no reply)	4	2	
**Symptom dimensions**	m (s)		
CP score	0.45 (2.62)	-1.75 (1.12)	t (80.9^b^)=-3.85 *p* < .001
CU score	0.48 (0.42)	0.26 (0.29)	t (45.6^b^)=-2.30 *p* = .024
Internalizing symptom score	9.91 (7.00)	6.60 (5.48)	t (104) = 22.02 *p* = .046
**ANS parameter**			
Resting state HF-HRV, ms^2^	1648.16 (1542.8)	1669.6 (1752.7)	t (103) = 0.05 ns (d = 0.01)
Resting state RMSSD, ms	61.84 (31.34)	63.29 (34.97)	t (103) = 0.18 ns (d = 0.03)
Gift bag HF-HRV, ms^2^	1740.1 (1889.6)	1962.1 (2558.2)	t (92) = 0.40 ns (d = 0.12)
Gift bag RMSSD, ms	62.33 (33.32)	63.96 (40.10)	t (92) = 0.17 ns (d = 0.06)
HF-HRV response (Gift bag – resting state), ms^2^	76.39 (932)	93.6 (1516)	t (89) = 0.06 ns (d = 0.02)
SCR, µS	57.63 (22.05)	67.50 (18.32)	t (36.8^b^) = 2.08 *p* = .051 (d = 0.42)
**Inhibitory control**	Mean rank	Mean rank	
Gift Bag score	54.16	66.09	Z=-1.99 *p* = .047

ADHD, attention deficit hyperactivity disorder; compl, completed; CP, conduct problems, symptoms of oppositional defiant disorder/ conduct disorder; CU, callous unemotional; d, effect size metric Cohen’s d; HF-HRV, high frequency heart rate variability; m mean, n number of cases; ns, not statistically significant; RMSSD, root mean square of successive differences; s standard deviation; SCR, skin conductance response;

^a^ Test statistics are reported for descriptive purposes. ^b^ Welch corrected df

### Variables

#### Comorbid symptoms

##### Conduct problems (CP) and CU traits

The conduct-disorder module of the CAPA was conducted with the mothers. Dimensional scores (sum of ODD and CD symptoms) from the CAPA were calculated. Additionally, the mother of the child filled in the German-language Parent Rating Scale for Oppositional Defiant and Conduct Disorder (FBB-SSV) (Döpfner and Görtz-Dorten 2017). From this questionnaire the oppositional symptoms scale, the conduct disorder symptoms scale, and the CU scale (low pro-social emotionality scale) were used. The questionnaire is suitable for the assessment of ODD/CD symptoms in line with the DSM-5 and ICD-10 and has shown good psychometric properties. In the following, we used a CP score (i.e. sum of the standardized ODD and CD symptom scores; Cronbach’s Alpha = 0.83) and a CU score (low pro-social emotionality score of the FBB-SSV).

##### Internalizing symptoms

The Anxious/Depressed scale of the German version of the Child Behavior Checklist (CBCL4–18) (Achenbach et al. 1991; Döpfner et al. 1994) was completed by the mothers. The scale is broadly used and shows good psychometric properties, for example significant associations with anxiety and affective disorders, indicating good validity (Döpfner et al. 1994).

#### Autonomic regulation

##### Procedure

ANS parameters were recorded during a lab session of about 20 min duration. After a familiarization period resting state vmHRV was recorded for 5 min. Thereafter the interview on attractive toys (Int-AT) task (description, see below) adapted from Asendorpf (1993) and the Gift-Bag task by Kochanska (2009) were conducted. The Gift-Bag task uses a delay of gratification (waiting) paradigm and captures reward-related inhibitory control. The child is told that he/she’ll receive a surprise, a present for participating in the study. After the Int-AT task and three minutes of waiting, the experimenter enters the room and places a bag (red paper gift bag) containing the gift for the child on the table in front of the child. The experimenter then leaves the room for five minutes (Gift Bag waiting period). The child is instructed not to look into the bag while awaiting the experimenter’s return with the mother.

##### vmHRV parameters

For comparison purposes we used frequency-domain (i.e. HF-HRV, reflecting the respiratory sinus arrhythmia, RSA) and time-domain measures (i.e. root mean square of successive differences, RMSSD) of vmHRV (Shaffer and Ginsberg 2017). Interbeat interval data was recorded via electrocardiogram (ECG) with Biopac Systems, Inc., MP150 Hardware. Sampling rate was set to 1000 Hz. Visual inspection has been conducted conscientiously by researchers. Kubios HRV Premium was used to process data. Kubios has an integrated R-wave detection algorithm and automatically bandpass filters the ECG. An automatic noise detection (set to medium in the present study) is included in the software. In the following, we use the vmHRV recordings of the 5 min resting state period and the 5 min waiting period of the Gift bag task and built a response score by subtracting the resting-state score from the waiting period score.

##### Sympathetic reactivity

Reactivity of the sympathetic nervous system can be validly measured by indices of electrodermal activity (EDA) (Boucsein et al. 2012). We measured the electrodermal reactivity to six questions from the Int-AT task. The task measures withdrawal vs. approach behavior and was adapted from Asendorpf (1993) (see also Pauli-Pott et al. 2023). The child is told that he/she will receive a gift for participating, but that before receiving the gift, he/she will take part in a video-recorded interview, conducted by a colleague, on the attractiveness of a series of toys. After three minutes of waiting, an unfamiliar adult enters the room, places six different toys in front of the child, and asks six questions, with a break of 10 s between the child’s answer and the next question. The procedure was videotaped, and video and EDA recordings were synchronized. The measurement of EDA followed the guidelines by Boucsein et al. (2012) using a BioPac MP150 system with two silver-silver chloride (Ag/AgCl) disposable electrodes attached to the middle phalanges of the middle and ring finger of the non-dominant hand. The frequency of the skin conductance responses (SCR; in microsiemens) elicited by the six questions was used as an indicator of the child’s sympathetic reactivity.

### Statistical analyses

The HF-HRV scores are treated as the primary measures of the vmHRV. RMSSD scores are used for comparison purposes. The HF-HRV measures (at baseline and during the waiting period of the Gift-Bag, delay of gratification task) were highly correlated (*r* > .80). We imputed single missing data (11.6%) of the Gift-Bag waiting period using a linear regression model. Associations of the HF-HRV scores with sex (*r* = .02 and 0.18), age of child (*r* = .14 and − 0.02), and maternal and paternal education level (r’s between − 0.02 and 0.15) were not statistically significant. We therefore refrained from adjusting analyses for influences of these variables.

The three hypotheses were tested using the multiple correlation coefficient between the symptom score and the two HF-HRV scores (i.e. resting-state, response). In post hoc analyses, the bivariate correlations (i.e. Pearson correlation coefficients and additionally non-parametric Spearman rank correlation coefficients) between symptom and HF-HRV scores were calculated. Correction for respiration rate was conducted. Each hypothesis is tested at a 5% level of significance.

In a descriptive analysis, associations between comorbid symptoms and the SCR score were calculated, and ADHD children were compared with healthy children in the HF-HRV scores.

## Results

The multiple correlation coefficient between CU traits and the two HF-HRV scores proved statistically significant (Table 2). Post hoc analyses revealed positive bivariate correlations of the CU score with the resting-state score and a marginally significant positive association with the response score. Children with higher CU traits showed higher HF-HRV resting-state and response scores (and vice versa). CP and internalizing symptoms were not significantly associated with the HF-HRV scores (Table 2).

**Table 2:** Correlations between comorbidity scores and vmHRV scores in children with ADHD

	CP score	CU score	Internalizing score
HF-HRV resting-state and Gift- Bag response score	**R = 0.15 ns**	**R = 0.30 *p*** **= .041**	**R = 0.06 ns**
Resting-state			
HF-HRV	− 0.02 (-0.02)	0.23t (0.26*)	0.06 (0.13)
HF-HRV corr.^1^	− 0.01	0.34**	0.06
RMSSD	0.01 (-0.03)	0.23t (0.29*)	0.15 (0.20)
Gift-Bag response			
HF-HRV	0.15 (0.23*)	0.23t (0.26*)	− 0.01 (0.02)
HF-HRV corr.1	0.16	0.23t	0.01
Gift-Bag raw score			
HF-HRV	0.06 (0.04)	0.30* (0.31*)	0.04 (0.09)
HF-HRV corr.^1^	0.08	0.39**	0.09
RMSSD	− 0.02 (0.02)	0.26* (0.27*)	0.10 (0.12)
SCR score	− 0.27* (-0.27*)	− 0.09 (-0.11)	− 0.21 (-0.25)

R, multiple correlation coefficient, in bold, tests of hypotheses;

Pearson correlations coefficients and Spearman rank correlation coefficients (in brackets) are reported; CP, conduct problems; CU callous unemotional traits; SCR, skin conductance response; statistical significance, t *p* < .10, * *p* < .05, ** *p* < .01; ns, not statistically significant;

^1^partial correlation coefficient corrected for respiration rate

The CP score was associated with the SCR score. The higher the CP were, the lower was the sympathetic response score (Table 2). Children with ADHD did not differ from healthy control children in the HF-HRV scores (Table 1). In all analyses, the use of RMSSD scores led to similar results.

## Discussion

vmHRV is regarded as a transdiagnostic marker of arousal/emotion regulation and cognitive control capacity. We analysed resting-state and task-related HF-HRV in a sample of psychotropic medication naïve children with ADHD. Based on previous theorizing and research, we expected to find associations of resting-state and task-related HF-HRV with internalizing symptoms, CP, and CU traits.

Consistent with expectations, CU traits related to the vmHRV scores in the children with ADHD. CP and internalizing symptoms were not associated with the vmHRV measures. CP related to low sympathetic reactivity. Consistent with previous research, no differences (small size of effects with d’s between 0.01 and 0.12) appeared between ADHD and healthy children in the vmHRV measures.

Our finding of a positive association between CU traits and HF-HRV (at rest and in response to the delay of gratification task) corresponds to findings by Thomson et al. (2020) and Goulter et al. (2019). Both studies found high vmHRV activity/reactivity to be associated with components of CU traits. Similarly, Fanti and Kimonis (2017) found that children with externalizing symptoms and high CU traits (and low internalizing symptoms) show better self-regulation capacity and less ANS arousal (i.e. lower heart rate) during resting state. These results correspond to the assumption of less impaired cognitive control capacity of children showing CP combined with CU traits compared to those with CP and low CU traits (Frick et al. 2014; Satlof-Bedrick et al. 2019). Hence, it might be tentatively speculated that among children with ADHD, high CU traits indicate less impaired self-regulation in the reward-related context.

CP did not relate to the vmHRV, but were associated with low sympathetic reactivity in the Int-AT task. The task elicits approach (attractive toys) versus withdrawal (being video-recorded while interviewed by an unfamiliar adult) behavior (see, Pauli-Pott et al. 2023). Relatively low sympathetic reactivity to anxiety eliciting stimuli of children with ODD/CD is well established (Fanti et al. 2019). This might underscore the validity of our result. We did not find an association between CU traits and the sympathetic reactivity. Recent studies revealed large individual differences in negative/anxious reactivity among children with CU traits. A primary CU trait variant with low anxiety has been distinguished from a secondary variant with high anxiety (Todorov et al. 2023; Craig et al. 2021; Docherty et al. 2016). It is well possible that our ADHD sample comprised both variants, and that therefore no overall association between CU traits and the sympathetic reactivity appeared.

We found no association between the vmHRV scores and internalizing symptoms. The finding contradicts our expectations and the results of meta-analyses which showed reduced vmHRV in depressive disorders (Koenig et al. 2016; Koch et al. 2019; Schiweck et al. 2019). The meta-analysis by Koenig et al. (2016) revealed that depressive symptoms do not relate to vmHRV in non-clinical, community-based samples of children and adolescents. Our result might be in line with this finding. Internalizing symptoms might not relate to vmHRV in psychotropic medication naive children with ADHD. However, the issue should to be further investigated in the future.

Our study has several strengths, such as the relatively large sample of medication naïve children with ADHD, the assessment of resting-state and task-related vmHRV, the additional use of a measure of the sympathetic reactivity, and the use of a small group of healthy children for comparison and validation purposes. Limitations of our study might be seen in the following issues. First, the distinction between CU traits and autism spectrum remains unclear. Consequently, an overlap between those constructs (Noppari et al. 2022; Leno et al. 2021), could potentially influence the results, despite the exclusion of children diagnosed with autism spectrum disorder from our study. Second, it would have been desirable to include further emotion regulation tasks (e.g. of anger/frustration) to assess the generalizability of results. Third, vmHRV measures represent economic, easily assessable indicators of adequate emotion regulation and adaptation to environmental demands. Nevertheless, peripheral ANS variables cannot be expected to precisely distinguish between brain-based bottom-up and top-down control processes over behavior. Fourth, the use of a longitudinal design would have been revealing to see whether vmHRV measures predict symptom development or vice versa.

To conclude, our result of relatively high vmHRV (at rest and during delay of gratification) of children with ADHD who show increased CU traits matches previous findings. These children showed more adaptive parasympathetic regulation. It might be tentatively speculated that increased CU traits indicate a subgroup of children with ADHD with a relatively less impaired self-regulation capacity in the reward-related context. The finding has to be further analyzed and confirmed by future studies. From a clinical perspective, a description of subgroups of children with ADHD regarding ANS functions and psychopathological symptoms seems worthwhile. As emotional, cognitive and ANS functions may affect treatment outcomes (Blair et al. 2014; Waller et al. 2013) precise description might help to adjust intervention strategies to meet the specific needs of these children.
